# Fast time-varying linear filters for suppression of baseline drift in electrocardiographic signals

**DOI:** 10.1186/s12938-017-0316-0

**Published:** 2017-02-07

**Authors:** Jiří Kozumplík, Ivo Provazník

**Affiliations:** Department of Biomedical Engineering, Brno University of Technology, Technická 12, 61200 Brno, Czech Republic

**Keywords:** Baseline drift, ECG signal, Time-varying linear filter

## Abstract

**Background:**

The paper presents a method of linear time-varying filtering, with extremely low computational costs, for the suppression of baseline drift in electrocardiographic (ECG) signals. An ECG signal is not periodic as the length of its heart cycles vary. In order to optimally suppress baseline drift by the use of a linear filter, we need a high-pass filter with time-varying cut-off frequency controlled by instant heart rate.

**Methods:**

Realization of the high-pass (HP) filter is based on a narrow-band low-pass (LP) filter of which output is subtracted from the delayed input. The base of an LP filter is an extremely low computational cost Lynn’s filter with rectangular impulse response. The optimal cut-off frequency of an HP filter for baseline wander suppression is identical to an instantaneous heart rate. Instantaneous length of heart cycles (e.g. RR intervals) are interpolated between QRS complexes to smoothly control cut-off frequency of the HP filter that has been used.

**Results and conclusions:**

We proved that a 0.5 dB decrease in transfer function, at a time-varying cut-off frequency of HP filter controlled by an instant heart rate, is acceptable when related to maximum error due to filtering. Presented in the article are the algorithms that enable the realization of time-variable filters with very low computational costs. We propose fast linear HP filters for the suppression of baseline wander with time-varying cut-off frequencies controlled by instant heart rate. The filters fulfil accepted professional standards and increase the efficiency of the noise suppression.

## Background

Heart frequency in humans can vary between around 0.67 to 3 Hz (40–180 beats/min) depending on age, sex, stress, health state and a number of other factors. The lower limit of the range can be found in only a small number of physically trained persons in rest, usually in supine position. The upper limit is usually reached only in extreme physical stress. Heart frequency is usually denoted as heart rate (HR) measured by the number of contractions of the heart/min.

**Fig. 1:**
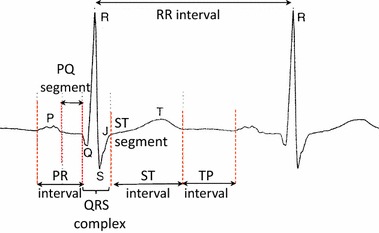
Main peaks (*Q*, *R*, *S*), waves (*T*, *P*), time intervals (*PR*, *ST*, *RR*) and segments (*PQ*, *ST*) in an ECG signal

Baseline wander is a noise with slow and usually large changes of the signal offset. Its frequency spectrum interferes with the frequency spectrum of the useful part of the signal—the ECG including its main waves and intervals: PR, ST, TP intervals, PQ segment, ST segment, and QRS complex (see Fig. 1). The main goal of filtering is to suppress the noise, while the useful signal cannot be distorted more than specified in a standard recommendation. If the ECG signal is (hypothetically) periodic, its first harmonic frequency would be identical with the heart frequency. Lower frequency components would only be composed of noise. Removing these components would not distort the shape of the ECG signal.

However, the ECG signal is not periodical but quasiperiodic (repetitive). Its heart frequency varies due to physiological or pathological reasons, thus it does not allow for the use of ideally set filters. Van Alsté et al. recommend attenuation of −0.5 dB at heart rate. In the case of on-line processing of longer signals, they recommend −0.5 dB at a fixed cut-off frequency 0.8 Hz [1]. The used filter may not introduce phase distortion. Cardiac electrophysiology societies recommend the use of a linear HP filter with cut-off frequency of 0.67 Hz and 3 dB attenuation. The AHA reports [2] and [3] recommend an amplitude response flat within <−0.5, 0.5> dB, within the range of 1.0–30 Hz. The reports recommend that low-frequency cut-off be 0.05 Hz to avoid possible distortion of ST segments, but this frequency can be relaxed up to 0.67 Hz (−3 dB) for linear digital filters with zero phase distortion. Abacherli et al. refers in [4] to standards which recommend an HP filter without phase distortion with −3 dB at 0.67 Hz to suppress baseline drift during monitoring. In diagnostic devices, standards recommend attenuation of −0.9 dB, at the same cut-off frequency of 0.67 Hz. Luo et al. refers in [5] to the same values and recommends attenuation not more than 0.5 dB at 1 Hz for stress-test ECG.

All mentioned recommendations and standards only deal with baseline wander suppression by linear filters with the fixed cut-off frequency. However, the main disadvantage of such filtering is that it sets a universal cut-off frequency which causes a lower efficacy in filtering ECG signals with a higher HR. It is generally known that baseline drift spectrum can significantly overlay spectrum of the useful part of ECG signals. Thus, it is desirable to use the highest possible cut-off frequency of the high-pass filter but acceptable regarding distortion of the useful part of ECG signals. This has been the reason for development of a number of alternative (non-linear) filtering methods.

Meyer et al. approximated baseline drift by generating cubic splines from knots in PR intervals where we expect zero line of the ECG signal [6]. The main disadvantage of this method was the necessity of PR interval detection. The method became more efficient with increasing HRs when we obtained higher density of knots, while useful parts of the signal remained uncorrupted.

Thakor et al. used a simple adaptive filter with a constant reference signal and a single weight [7]. However, this filtering method was a source of certain ST segment distortion. Jane et al. [8] described a method based on a cascade of two adaptive filters. The first, simple, adaptive filter with a constant reference input and a single weight represented a simple HP filter with cut-off frequency of about 0.3 Hz. Its output fed a QRS complex detector that produced impulses derived from a rhythm of detected QRS complexes. The impulses entered the reference input of the second adaptive filter with a number of weights equal to a number of samples of the ECG cycle. The filter suppressed signals not correlated with the useful part of the ECG signal. ST segments were not distorted thanks to their direct relation to QRS complexes. A cascade adaptive filter was also used by Laguna et al. [9].

Blanco-Velasco et al. exploited methods based on empirical mode decomposition (EMD) [10]. EMD decomposed the signal on a sum of intrinsic mode functions. These were derived directly from an analysed signal and represented a simple oscillatory mode as a counterpart to the simple harmonic function used in Fourier analysis.

Shusterman et al. developed a two-step procedure to correct baseline drift [11]. Firstly, two infinite impulse response filters were applied in a backward and forward direction to avoid phase distortion and obtained ECG signals free of large baseline wander. Secondly, QRS complexes were detected and the rest of the baseline drift was interpolated from determined PQ and TP intervals.

Shin et al. used modified non-linear methods originally designed for the detrendization of heart rate variability signals to suppress baseline drift [12]. The resulting trend was derived from an estimation of overlapping short-time trends and was based on a smoothness prior approach.

Fasano et al. applied an approach of baseline wander estimation and its removal in ECG signals based on the approximation of quadratic variation (measure of variability for discrete signals) reduction. Baseline wander was estimated by solving a constrained convex optimization problem where quadratic variation entered as a constraint [13].

Sharma et al. [14] described a method based on Hilbert vibration decomposition. The method considered the first component of the decomposition when applied to an ECG signal that corresponds to baseline wander of the signal.

Zivanovic et al. introduced a baseline wander modelling using low-order polynomials [15].

Hao et al. designed in [16] filtering based on an estimation of baseline wander using the mean–median filter and discrete wavelet transform.

This paper presents an application of a linear filter with a time-varying impulse response. This allows us to fulfil accepted professional standards and to increase the efficiency of the noise suppression. The main aim is to reach a maximum possible attenuation based on an instant HR.

Linear filters provide the correct filtering and it is widely accepted by the biomedical engineering community. At the same time, this filter cannot be considered as optimal due to its variable heart frequency. For more effective suppression of baseline drift, an HP filter with time-varying cut-off frequency related to instant heart frequency should be used.

Sörnmo proposed in [17] and [18] a time-varying filter. In [17], he used a bank of low pass filters with cut-off frequencies 0.5, 0.75, 1.0, 1.25 a 1.5 Hz (at −6 dB), the output of the filters were subtracted from the delayed input signal. Selection of a filter from the bank was based on the length of RR interval, or estimation of drift. Sampling frequency was decimated from 500 to 12.5 Hz to decrease computational cost of the filtering. However, decimation and interpolation caused a higher phase delay of the filter.

We propose a time-varying linear HP filter which does not introduce any phase distortion and excels with an extremely low computational load. The frequency response of the filter is adapted to an instant (interpolated) HR in each signal sample.

## Methods

### Filter design

Linear phase frequency characteristics beginning at the origin of axes of the phase frequency response are a strict requirement to prevent phase distortion that could decline the ST segment. This requirement can be fulfilled by using a finite impulse response (FIR) linear filter with symmetric impulse response.

The considered filters are a relatively narrow-band; thus their impulse responses are relatively long (up to hundreds samples). Direct realization of classical FIR filters leads to a high load of signal response computation which is not mainly suitable in real time applications incorporating signal processors. Low computational costs can be achieved by an elegant solution employing Lynn’s LP filters. These are called simple moving-average filters with a rectangular impulse response [19]. Realization of the required HP filter *H*_*HP*_ is based on a narrow-band LP filter *H*_*LP*_ of which output is subtracted from the delayed input

1 $$H_{HP} \left( z \right) = z^{ - \tau } \text{ $-$ }H_{LP} \left( z \right).$$

Lynn’s LP filter is a comb filter with *N* zeroes uniformly positioned on the unit circle in *z*-plain. The first zero is at *z* = 1. The LP filter is constructed by inserting a single pole to *z* = 1. It results in a recursive FIR filter *G* with rectangular impulse response. Its transfer function is

2 $$G\left( z \right) = \frac{{z^{N} - 1}}{{Nz^{N - 1} \left( {z - 1} \right)}} = \frac{{1 - z^{ - N} }}{{N\left( {1 - z^{ - 1} } \right)}}.$$

The filter may be described in its non-recursive form with the transfer function *H*

3 $$H\left( z \right) = \left( {1 + z^{ - 1} + z^{ - 2} + \cdots + z^{{ - \left( {N - 1} \right)}} } \right)/N.$$

**Fig. 2:**
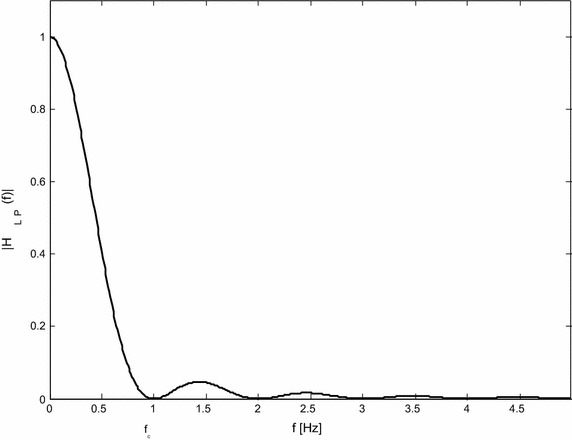
Example of a cascade of two identical Lynn’s LP filters. The amplitude frequency response *G*_*LP*_ (*H*_*LP*_) for *fs* = 500 Hz, *N* = 500, and *f*_*c*_ = *fs*/N = 1 Hz

Lynn’s LP filter as defined by (2) has a high stop-band ripple. Thus, it is recommended to use a cascade of two identical filters with transfer function *G*_*LP*_ (see Fig. 2).

4 $$G_{LP} \left( z \right) = G\left( z \right)G\left( z \right) = \left( {\frac{{1 - z^{ - N} }}{{N\left( {1 - z^{ - 1} } \right)}}} \right)^{2} .$$

Module of the transfer function *G*_*HP*_ has an acceptable passband ripple from 0.0 to −0.4 dB according to [2]. Module of transfer function *G*_*HP*_ reaches 1 at *f*_*s*_/*N*, where *f*_*s*_ is the sampling frequency.

The cascade *G*_*LP*_ can be realized in a non-recursive form with transfer function *H*_*LP*_.

5 $$H_{LP} \left( z \right) = H\left( z \right)H\left( z \right) = \left( {1 + 2z^{ - 1} + \cdots + Nz^{{ - \left( {N - 1} \right)}} + \cdots + 2z^{{ - 2\left( {N - 1} \right) - 1}} + z^{{ - 2\left( {N - 1} \right)}} } \right)/N^{2}.$$

Both the recursive and non-recursive realizations of the cascade of two identical filters *G*_*LP*_, or *H*_*LP*_ respectively, have a triangular impulse response.

The fundamental frequency of an idealized periodic ECG signal is

6 $$f_{ECG} = \frac{1}{{\left( {N_{RR} - 1} \right)T_{S} }},$$

where *N*_*RR*_ is a number of samples of an ECG cycle that ideally has a constant length, and *T*_*S*_ is a sampling period. When module frequency response of an HP filter is expected to be 1 at frequency *f*_*ECG*_, then

7 $$N_{RR} = \frac{{f_{S} }}{{f_{ECG} }} + 1,$$

where *f*_*s*_ is a sampling frequency. If $f_{S} > > f_{ECG} ,$ then

8 $$N = round\left( {\frac{{f_{S} }}{{f_{ECG} }}} \right) \approx N_{RR}.$$

Thus, *N* can be directly derived from a number of samples of a RR interval provided that the RR interval represents the ECG cycle. A number of samples of the symmetric impulse response of the HP filter realized using a cascade of two identical LP filters and subtraction are always odd

9 $$N_{HP} = 2N - 1 ,$$

and the phase delay of the HP filter is an integer

10 $$\tau_{HP} = \frac{{N_{HP} - 1}}{2} = N - 1.$$

In this case, the module frequency response value will be 1 at frequency $f_{C} \approx f_{ECG}$. If we require the filter gain to be equal to −0.5 dB at the frequency $f_{C}$ (transfer 0.9441), we need to decrease the value of $N$ that leads to widening the stop-band of the HP filter. Considering that $N$ corresponds to the frequency *f*_*C*_ = *f*_*ECG*_ for zero gain decrease, the required value of *N*_*C*_ at frequency *f*_*C*_ for 0.5 dB gain decrease is computed by multiplication or division by an appropriate constant.

**Fig. 3:**
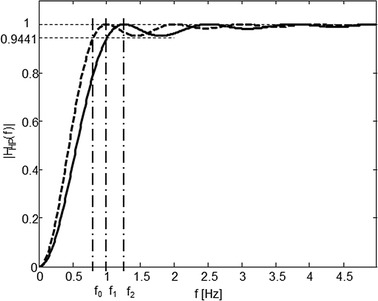
Graphical representation of the ratio of a couple of frequencies with transfers 1 and 0.9441 (−0.5 dB). The amplitude frequency response *G*_*HP*_ (*H*_*HP*_) of the derived HP filter *G*_*HP*_(*z*) = *z* ^−*τ*^ *−* *G*_*LP*_(*z*) for *fs* = 500 Hz and *f*_*c*_ ≈ 1 Hz

As we can consider the ratio of two frequencies with transfers 1 and 0.9441 (−0.5 dB) constant, we can write according to Fig. 3

11 $$c = \frac{{f_{1} }}{{f_{0} }} = \frac{{f_{2} }}{{f_{1} }} \to f_{2} = cf_{1} .$$

The constant *c* can be evaluated as follows. The high-pass filter *H*_*LP*_ is derived from a low-pass filter with recursive realization described by (4). Its amplitude frequency response *G*_*LP*_ is

12 $$\left| {G_{LP} \left( {e^{{j\omega T_{s} }} } \right)} \right| = \left| {\frac{{1 - e^{{ - j\omega T_{s} N}} }}{{N\left( {1 - e^{{ - j\omega T_{s} }} } \right)}}} \right|^{2} = \left| {\frac{{e^{{ - j\omega T_{s} N/2}} \left( {e^{{j\omega T_{s} N/2}} - e^{{ - j\omega T_{s} N/2}} } \right)}}{{Ne^{{ - j\omega T_{s} /2}} \left( {e^{{j\omega T_{s} /2}} - e^{{ - j\omega T_{s} /2}} } \right)}}} \right|^{2} = \left| {\frac{{sin\left( {\omega T_{s} N/2} \right)}}{{Nsin\left( {\omega T_{s} /2} \right)}}} \right|^{2}.$$

For *ω* = *ω*_*c*_

13 $$\omega T_{S} N = 2\pi N\frac{{f_{c} }}{{f_{s} }} = 2\pi \frac{{f_{c} }}{{f_{0} }}.$$

Then

14 $$\left| {\frac{{sin\left( {\pi \frac{{f_{c} }}{{f_{0} }}} \right)}}{{Nsin\left( {\pi \frac{{f_{c} }}{{f_{s} }}} \right)}}} \right|^{2} = 0.0559,$$

where 0.0559 is transfer of a low-pass filter *G*_*LP*_ (*H*_*LP*_) at *f*_*c*_ and corresponds to transfer 0.9441 of a high-pass filter *H*_*HP*_ at *f*_*c*_ = *f*_*ECG*_ (gain equals to −0.5 dB).

As *f*_*c*_ ≪ *f*_*s*_, we can write

15 $$\left| {\frac{{sin\left( {\pi \frac{{f_{c} }}{{f_{0} }}} \right)}}{{\pi \frac{{f_{c} }}{{f_{0} }}}}} \right|^{2} \cong 0.0559.$$

We can easily derive that $\frac{{f_{c} }}{{f_{0} }} = c = 1.253$.

As the cut-off frequency and the length of the impulse response are inversely related, we can write

16 $$N_{c} = \frac{N}{c} \approx round\left( {\frac{N}{1.253}} \right).$$

### Fixed filter realization

Presented above was the idea of an optimal HP filter with its impulse response length controlled by the instant length of an ECG cycle. Such a filter has a maximum possible attenuation in a frequency band below *f*_*ECG*_ that can be reached by a linear system of this type. Further, the proposed filter is linear and it has linear phase frequency characteristics that are required for the processing of ECG signals.

Recursive realization of the Lynn’s filter is not an appropriate solution. Although the single pole on a unit circle counteracts with a zero at the same position, there are rounding errors due to division by a large number *N* ^*2*^. This negatively influences filtration.

Non-recursive realization of the convolution leads to large impulse responses, thus it can be computationally expensive and slow. However, non-recursive realization can be represented by a cascade of two non-recursive (moving-average) filters with a low number of necessary operations per sample interval. The idea is based on the use of a filter *H* with a rectangular impulse response where we add a new input sample to a sum, then we subtract the oldest input sample and finally divide by a constant *N* in each sampling interval. Two such filters in a series represent an LP filter with triangular impulse response. The needed HP filter requires one more subtraction.

The realized filter represents a fixed system based on Lynn’s filter with a low number of required operations. Its cut-off frequency can be chosen in advance. However, such a solution is the appropriate basis to design an elegant filter with a time-varying impulse response (and thus time-varying cut-off frequency).

### Time-varying impulse response filter realization

An ECG signal is not periodic—the length of its heart cycle(s) vary. To suppress baseline drift optimally, we need an HP filter with time-varying cut-off frequency controlled by an instant HR. The heart frequency in each time instant can only be estimated as we usually measure heart cycles from detected QRS complexes. However, the instant length of heart cycles (e.g. RR intervals) can be interpolated to obtain a signal *N*_*RR*_(*n*) to smoothly control the cut-off frequency of the HP filter being used. We use simple 1^st^ order interpolation (by a line).

Fundamental frequency of the ECG signal is then varying

17 $$f_{ECG} \left( n \right) = \frac{1}{{\left( {N_{RR} \left( n \right) - 1} \right)T_{S} }}.$$

When the module frequency response of an HP filter is expected to be equal to 1 at frequency *f*_*ECG*_(*n*), then the number of samples of the rectangular impulse response in *n*-th cycle is

18 $$N\left( n \right) = round\left( {\frac{{f_{S} }}{{f_{ECG} \left( n \right)}}} \right).$$

Thus, we can compute *N*(*n*) for each *n* directly from interpolated values of RR intervals. In other words, we design a new LP filter that always has an odd number of impulse response samples *N*_*LP*_(*n*) for each *n* by the above simple procedure

19 $$N_{LP} \left( n \right) = 2N\left( n \right) - 1.$$

The impulse response is triangular; its values can be easily derived.

### Direct realization of an LP filter with minimum delay

The designed HP filter must possess a constant phase delay despite the time-varying length of its impulse response. Therefore, the phase delay *τ* of the final HP filter is adapted to the maximum desirable delay that corresponds to the longest expected RR interval. The longest expected RR interval is derived from the lowest expected heart rate 40 beats/min (i.e. 0.67 Hz) [2, 3].

20 $$\tau = \frac{{N_{{HP_{max} }} - 1}}{2} = N_{max} - 1.$$

Interpolated instant values of RR intervals are stored in a circular buffer that contains *N*_*max*_ samples corresponding to the longest possible impulse response of the Lynn’s filter.

The transfer function of the LP filter for current *N* in each *n*

21 $$H_{LP} \left( z \right) = z^{ - \tau } H\left( z \right)H\left( z \right) = z^{{ - \left( {N_{max} - 1} \right)}} \left( {z^{ - 1} + 2z^{N - 2} + \cdots + N + \cdots + 2z^{{ - \left( {N - 2} \right)}} + z^{{ - \left( {N - 1} \right)}} } \right)/N^{2}.$$

It is obvious from (17) that the LP filter impulse response has always an odd number of samples.

The corresponding difference equation in non-casual form for *l* = *n* *−* *τ* is

22 $$y_{LP} \left( l \right) = \left[ {x\left( {l + N - 1} \right) + 2x\left( {l + N - 2} \right) + \cdots + Nx\left( l \right) + \cdots + 2x\left( {l - N + 2} \right) + x\left( {l - N + 1} \right)} \right]/N^{2} ,$$

where we used *N* = *N*(*l*) = *N*(*n* *−* *τ*) for simplicity of equational notation.

**Fig. 4:**
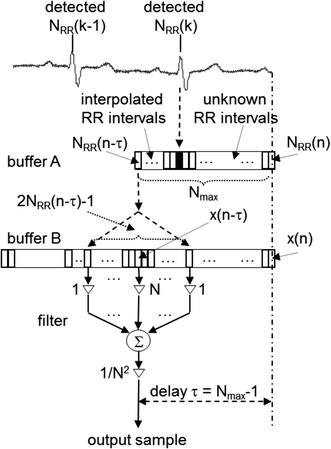
Schematic representation of direct realization of the LP filter with minimum delay. *Buffer A* buffer of RR intervals (*N*_*max*_ length), *buffer B* buffer of the input signal samples (2*N*_*max*_ − 1 length), *filter* a filter with impulse response *h*(*n*) = {1, 2, 3,…, *N*,…, 3, 2, 1}, *N*_*RR*_ number of sampling intervals, *N*_*RRmax*_ number of samples of the longest expected RR interval, *x*(*n*) current input sample

The principle of computation of the output sample is presented in Fig. 4. We should note that if *N*(*n*) varies with time, the impulse response can be gradually extended or shortened with a minimum step of two samples to keep its symmetry along the middle sample.

Direct realization of the LP filter with the triangular impulse response with 2*N* − 1 samples (see Fig. 4) has no advantage of low computational complexity due to constantly changing all weights of the filter in time.

### Realization of an LP filter by a cascade of two Lynn’s filters (knot inside QRS complexes)

**Fig. 5:**
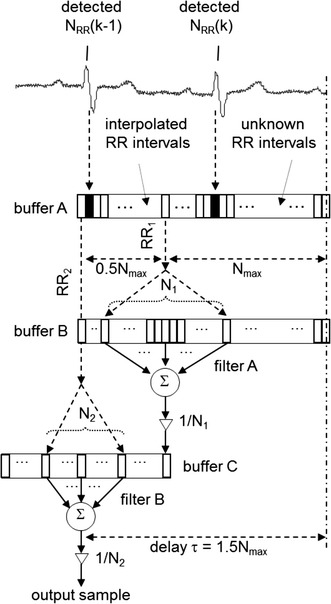
Schematic representation of realization of the LP filter by a cascade of two Lynn’s filters with knots inside QRS complexes. *Buffer A* a buffer of RR intervals (1.5*N*_*max*_ length), *buffer B* a buffer of the input signal samples (1.5*N*_*max*_ length), *buffer C* a buffer of the output signal from filter A (*N*_*max*_ length), *filter A* a filter with impulse response ^*1*^ *h*(*n*) = {1, 1, 1,…, 1}, *filter B* a filter with impulse response ^*2*^ *h*(*n*) = {1, 1, 1,…, 1}, *N*_*1*_ and *N*_*2*_ odd numbers

Using a cascade of two LP filters is more beneficial because both filters in a series have the same rectangular impulse responses (see Fig. 5). A new sample is added if we consider a fixed length of the impulse response and the oldest sample is subtracted from a sum in each cycle. Under the condition that both impulse responses must be symmetrical along their middle sample (as required for integer delay of the final filter), i.e. N must be odd, the impulse response of each filter will vary with a minimum step of two samples. This results in a minimum step of four samples for two filters in a series.

We need to use a buffer of input signal samples (input for the first filter) and a buffer of first filters output samples (input for the second filter) besides a buffer of values of RR intervals.

The maximum length of the impulse response of each of the used filters is equal to *N*_*max*_. Delay of the first filter must also be *N*_*max*_ to be able to interpolate all needed values of the longest possible RR interval. Total delay of the final LP filter (as well as the HP filter) is.

23 $$\tau = 1.5N_{max}.$$

### Realization of an LP filter by a cascade of two Lynn’s filters (knots between QRS complexes)

Impulse responses of LP filters can vary in time differently based on how we interpolate RR intervals. Intuitively, we could place knots in the middle between neighbour QRS complexes, instead of placing them into QRS complexes as described in part “Realization of an LP filter by a cascade of two Lynn's filters (knot inside QRS complexes” section of methods.

**Fig. 6:**
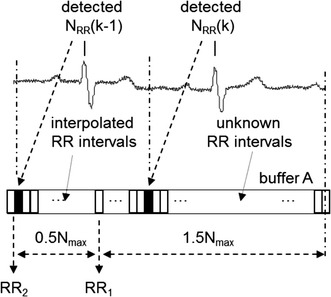
Schematic representation of RR interval interpolation for the LP filter realized by a cascade of two Lynn’s filters with knots between QRS complexes. *Buffer A* a buffer of RR intervals (2*N*_*max*_ length)

Then the buffer with interpolated values of RR intervals must be longer by a half of the longest expected RR interval (see Fig. 6). Thus total delay of the final filter will increase to.

24 $$\tau = 2N_{max}.$$

## Results

### Computational complexity

The algorithm realizing the final filter provides interpolation of RR intervals and computation of the output sample that contribute to total computational load.

We need to determine a step Δ_*RR*_ after detecting a *k*-th QRS complex, i.e. deduction of *N*_*RR*_(*k*) to interpolate RR intervals.

25 $$\Delta_{RR} = \frac{{N_{RR} \left( k \right) - N_{RR} \left( {k - 1} \right)}}{{N_{RR} \left( k \right)}}.$$

The step Δ_*RR*_ will be successively added to the previous value *N*_*RR*_(*k* − 1). In each cycle of computation of the output signal sample, we can compute interpolated value of the RR interval by adding value of *round*(*m*Δ_*RR*_) to the current value. Index *m* is defined as *m* = 1, 2, …, *N*_*RR*_(*k*) − *N*_*RR*_(*k* − 1).

The complexity of computation of output samples of the used LP filters depends on how *N* varies. For each filter, we need to add one sample value and to subtract one sample value if *N* is constant. For varying *N*, we will add and subtract two samples at maximum, because it applies.

26 $$\left| {\Delta_{RR} } \right| = \left| {1 - \frac{{N_{RR} \left( {k - 1} \right)}}{{N_{RR} \left( k \right)}}} \right| \le 2.$$

Both LP filters also require single division by a current number of samples of a corresponding impulse response. The final HP filter requires one more subtraction of LP filter output from a delayed input signal.

The advantage of the proposed algorithm lies in the extremely fast computation of its response due to simplicity of the used filter. As mentioned in the part Computational complexity in "Results" section, the filter requires 6 additions (or subtractions, respectively) and 2 divisions only to compute one output signal sample. Extremely low computational demands together with the highest possible efficiency of baseline wander suppression regarding to instant heart rate favour the proposed filter against the other time-varying systems presented in “Background” section. One of the most advanced adaptive filter to suppress baseline wander was presented in [17]. However, the used bank of low pass filters requires simultaneous computation of responses of many filters in order to deliver smooth output signal when switching between filters. Further, decimation and interpolation filters are never ideal and they are sources not only of higher phase delay but also of errors.

The algorithms were tested on MA1 set signals from The common standards for electrocardiography (CSE) database [20]. The signals were of 10 s length, sampled at *f*_*s*_ = 500 Hz with quantization step 5 µV (4.8828125 µV). Artificial signals of CSE database were derived from real signals with common noise (without baseline wander) and periodized. The spectrum of each artificial signal is discrete, the first spectral line is located at the signal’s fundamental frequency *f*_*ECG*_. The signals do not contain any baseline drift. Thus, a linear HP filter with transfer = 1 at *f*_*ECG*_ does not distort the signal. Hence, the MA1 signals were ideal for evaluation of signal distortion due to application of an HP filter with cut-off frequency equal to instant *f*_*ECG*_. The higher attenuation of the filter allows for more efficient suppression of the drift concerning its spectrum is usually partially overlapped with the lower spectrum of the useful signal.

**Fig. 7:**
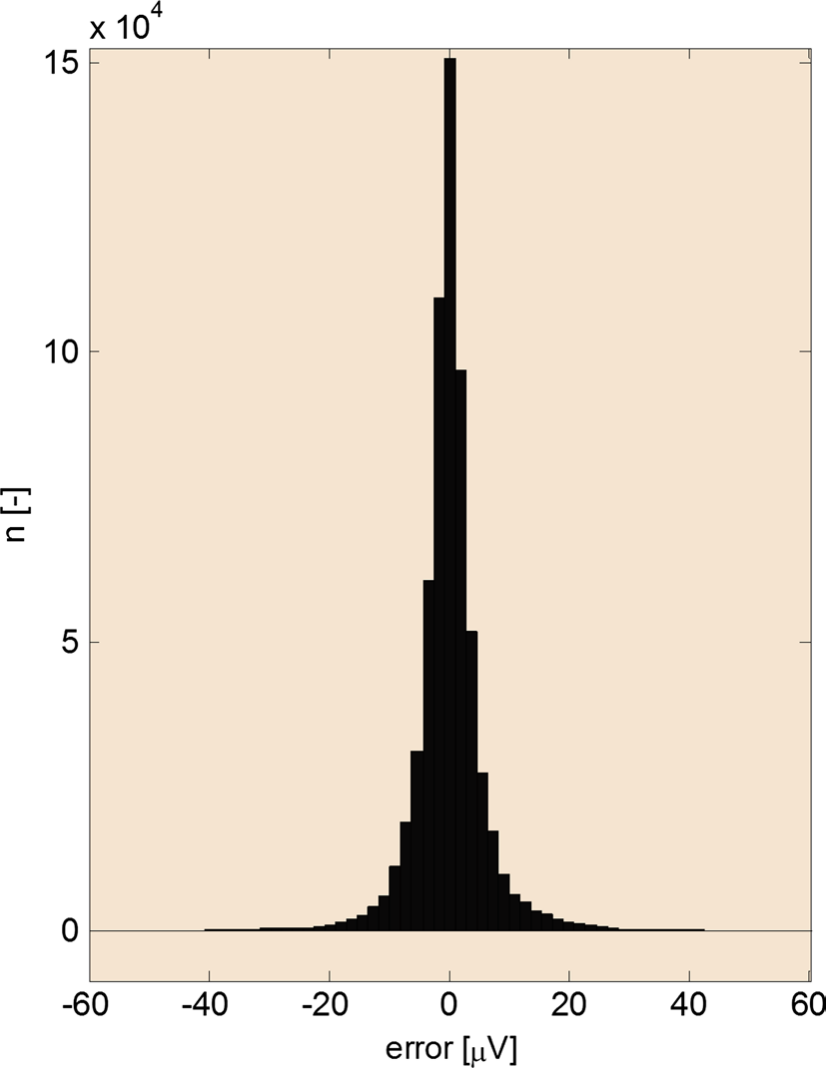
Histogram of errors after filtering with HP filter with attenuation −0.5 dB at cut-off frequency equal to heart frequency

A set of 125 12-lead (1500 in total) artificial signals MA1 of the CSE database with constant RR intervals were chosen for testing. We evaluated distortion after filtering with a linear HP filter caused by various attenuations at cut-off frequency equal to heart frequency *f*_*ECG*_. As a compromise, we accepted cut-off frequency for attenuation by 0.5 dB at *f*_*ECG*_. Figure 7 show a histogram of errors in all tested signals filtered by such a filter. The histogram includes only values of a single cycle of each periodic signal. The resulting mean error is 0.0124 µV with standard deviation 6.1418 µV. The value of standard deviation is comparable to the quantization step of the input signals. Attenuation by 0.5 dB corresponds to transfer 0.9441 so that the used HP filter decreases amplitude of the first harmonic by 5.6%.

**Fig. 8:**
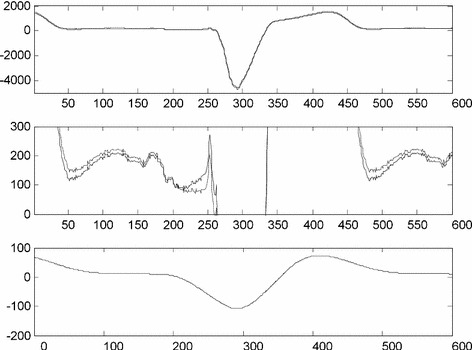
Input signal No. MA1_065_12 (lead V2) x(n) and output signal y(n) are visually identical in standard scale (*upper panel*) for the time-varying HP filter with −0.5 dB at *f*_*c*_ = *f*_*ECG*_. Vertical detail of x(n) (*light grey line*) and y(n) (*black line*) (*middle panel*). Error signal e(n) = x(n) − y(n) (*lower panel*)

The highest error for attenuation −0.5 dB at cut-off frequency were found in lead V2 of signal No. MA1_065_12. The result is depicted in Fig. 8. Such high error is caused by an unusually high S-wave (−4.7 mV) and T-wave (1.5 mV). Figure 8 (middle panel) shows a distortion of low R-wave and its neighbourhood. T-wave peak has been decreased by 71 µV (about 5%) and S-wave peak by 107 µV (about 2%).

## Discussion

**Fig. 9:**
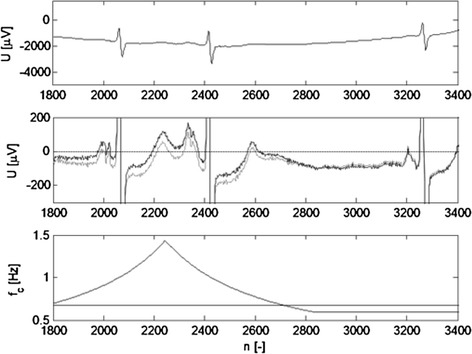
Input signal No. MO1_023_12 (lead V3) (*upper panel*). Vertical detail of HP output for *f*_*c*_ = 0.67 Hz (−0.5 dB) (*light grey line*) and time-varying HP output (−0.5 dB at *f*_*c*_ = *f*_*ECG*_) with knots between QRS complexes (*black line*) (*middle panel*). Constant (0.67 Hz) and time-varied HP cut-off frequency (*lower panel*)

Real ECG signals show a time-varying heart frequency; thus the signal is not periodic. Actual length of the period (ECG cycle) can be measured in non-equidistant knots only—i.e. at the points where QRS complexes are identified. The idea of a time-varying filter considers the fact that the period length does not change suddenly when a new QRS complex is detected. Thus, cut-off frequency of the designed HP filter changes gradually. At each time instant, linear interpolation is applied in between neighbouring RR intervals derived from QRS detection. Then the actual length of an RR interval is computed at each time instant, i.e. between QRS complex detection points. Instant heart frequency (and thus cut-off frequency of the filter) is estimated as reverse value of RR interval estimation. Figure 9 shows an example of baseline drift suppression in a real ECG signal No. MO1_023_12 (lead V3) from CSE database.

The method introduced for suppression of baseline drift in ECG signals using a linear time-varying HP filter represents optimal linear filtering with regard to setting its cut-off frequency. The cut-off frequency is controlled with instant (interpolated) heart frequency; thus the main disadvantage of a traditional linear filter in this application is the necessity of using a fixed cut-off frequency while the heart frequency physiologically varies. As it is well known, the fixed cut-off frequency is set to a certain value. This is in order to reach a maximum allowed distortion of the useful part of the signal under the worst conditions. Such an approach must be based on the lowest considered heart frequency. However, a more efficient baseline wander suppression requires a higher cut-off frequency in most cases. We proved that a 0.5 dB decrease in transfer function at cut-off frequency is acceptable when related to maximum error due to filtering.

The presented filter was evaluated by testing on a set of ECG signals of standard CSE database. The resulting mean error and standard deviation was low at the level of quantization step of the input signals.

The proposed method depends on reliable detection of QRS complexes. However, a QRS complex detector is a standard basic part of all ECG processing systems and its output is used for pre-processing and delineation of ECG signals. Impact of false positive or false negative detections of heart cycles on the filter efficacy is as follows. When any QRS complex is missed by the detector, only the length of the filter is effected and its cut-off frequency is decreased. Baseline wander removal may be less efficient, the useful part of the processed ECG signal is not distorted. When false QRS complex is detected (false extra heart beat “found”), cut-off frequency of the filter increases by shortening its length. Baseline wander removal is more efficient. However, the useful part of the processed ECG signal is not distorted if we prevent the situation by setting minimum length of the filter to highest expected heart rate. The highest expected rate has to be set according to clinical application: rest electrocardiography, stress test electrocardiography, etc.

## Conclusion

A linear time-varying HP filter for optimal suppression of baseline drift was presented. The filter controls its cut-off frequency using an estimation of an instant HR. Such an approach allows us to reach the maximum possible attenuation of the filter while accepted professional standards on maximum allowed distortion are fulfilled. Further, there is no need to set a fixed cut-off frequency that would limit the highest possible frequency of a passband. The filter is suitable for standard ECG devices but also for smart/wearable solutions due to its simplicity and low computational demands.

## Abbreviations

LP: low-pass
HP: high-pass
ECG: electrocardiography
HR: heart rate
CSE: Common Standards for Electrocardiography

## References

[1] Van Alsté JA, Schilder TS (1985). Removal of base-line wander and power-line interference from the RCG by an efficient FIR filter with a reduced number of taps. IEEE T Bio-Med Eng..

[2] Bailey JJ, Berson AS, Garson A, Horan LG, Macfarlane PW, Mortara DW, Zywietz C (1990). Recommendations for standardization and specifications in automated electrocardiography—bandwidth and digital signal-processing—a report for health-professionals by an ad hoc writing group of the committee on electrocardiography and cardiac electrophysiology of the council-on-clinical-cardiology. Am-Heart-Assoc Circ.

[3] Kligfield P, Gettes LS, Bailey JJ, Childers R, Deal BJ, Hancock EW, van Herpen G, Kors JA, Macfarlane P, Mirvis DM, Pahlm O, Rautaharju P, Wagner GS (2007). Recommendations for the standardization and interpretation of the electrocardiogram—Part I: the electrocardiogram and its technology—a scientific statement from the American Heart Association Electrocardiography and Arrhythmias Committee, Council on Clinical Cardiology; the American College of Cardiology Foundation; and the Heart Rhythm Society—Endorsed by the International Society for Computerized Electrocardiology. Circulation.

[4] Abacherli R, Schmid HJ (2009). Meet the challenge of high-pass filter and ST-segment requirement with a DC-coupled digital electrocardiogram amplifier. J Electrocardiol.

[5] Luo S, Johnston P (2010). A review of electrocardiogram filtering. J Electrocardiol.

[6] Meyer CR, Keiser HN (1977). Electrocardiogram baseline noise estimation and removal using cubic-splines and State-space computation techniques. Comput Biomed Res.

[7] Thakor NV, Zhu Y-S (1991). Applications of adaptive filtering to ECG analysis: noise cancellation and arrhythmia detection. IEEE T Bio-Med Eng..

[8] Jane R, Laguna P, Thakor NV, Caminal P. Adaptive baseline wander removal in the ECG: comparative analysis with cubic spline technique. Comput Cardiol. 1992:143–6.

[9] Laguna P, Jané R, Caminal P. Adaptive filtering of ECG baseline wander. In: 14th Annual International Conference of the IEEE EMBS, conference proceedings. Engineering in Medicine and Biology Society; 1992, p. 509–10.

[10] Blanco-Velasco M, Weng BW, Barner KE (2008). ECG signal denoising and baseline wander correction based on the empirical mode decomposition. Comput Biol Med.

[11] Shusterman V, Shah SI, Beigel A, Anderson KP (2000). Enhancing the precision of ECG baseline correction: selective filtering and removal of residual error. Comput Biomed Res.

[12] Shin SW, Kim KS, Song CG, Lee JW, Kim JH, Jeung GW (2015). Removal of baseline wandering in ECG signal by improved detrending method. Bio-Med Mater Eng.

[13] Fasano A, Villani V (2014). Baseline wander removal for biological signals by qudratic variation reduction. Signal Process..

[14] Sharma H, Sharma KK (2015). Baseline wander removal of ECG signals using Hilbert vibration decomposition. Electron Lett.

[15] Zivanovic M, González-Izal M (2013). Simultaneous powerline interference and baseline wander removal from ECG and EMG signals by sinusoidal modeling. Med Eng Phys.

[16] Hao W, Chen Y, Xin Y. ECG Baseline wander correction by mean-median filter and discrete wavelet transform. In: 33rd Annual International Conference of the IEEE EMBS, conference proceedings. Engineering in Medicine and Biology Society, Boston, MA, USA; 2011. p. 2712–15. 10.1109/IEMBS.2011.6090744 22254901

[17] Sörnmo L (1993). Time-varying digital filtering of ECG baseline wander. Med Biol Eng Comput..

[18] Sörnmo L, Laguna P (2005). Bioelectrical signal processing in cardiac and neurological applications.

[19] Lynn PA, Fuerst W (1992). Introductory digital signal processing with computer applications.

[20] Willems J, Arnaud P, van Bemmel JH, Bourdillon PJ, Degani R, Denis B, Harms FMA, Macfarlane PW, Mazzocca G, Meyer J, van Eck HJR, de Medina EOR, Zywietz C (1985). Establishment of a reference library for evaluating computer ECG measurement programs. Comput Biomed Res.

